# Development and optimization of human deuterium MR spectroscopic imaging at 3 T in the abdomen

**DOI:** 10.1002/mrm.30556

**Published:** 2025-05-20

**Authors:** Mary A. McLean, Ines Horvat Menih, Pascal Wodtke, Joshua D. Kaggie, Jonathan R. Birchall, Rolf F. Schulte, Ashley Grimmer, Elizabeth Latimer, Marta Wylot, Maria J. Zamora Morales, Alixander S. Khan, Huanjun Wang, James Armitage, Thomas J. Mitchell, Grant D. Stewart, Ferdia A. Gallagher

**Affiliations:** ^1^ Department of Radiology University of Cambridge Cambridge UK; ^2^ Cancer Research UK Cambridge Centre Cambridge UK; ^3^ GE HealthCare Munich Germany; ^4^ Department of Urology, Addenbrooke's Hospital Cambridge University Hospitals NHS Foundation Trust Cambridge UK; ^5^ Department of Surgery University of Cambridge Cambridge UK

**Keywords:** deuterium metabolic imaging, heavy water, kidney, liver, metabolism, oncocytoma

## Abstract

**Purpose:**

To establish and optimize abdominal deuterium MR spectroscopic imaging in conjunction with orally administered ^2^H‐labeled molecules.

**Methods:**

A flexible transmit‐receive surface coil was used to image naturally abundant deuterium signal in phantoms and healthy volunteers and after orally administered ^2^H_2_O in a patient with a benign renal tumor (oncocytoma).

**Results:**

Water and lipid peaks were fitted with high confidence from both unlocalized spectra and from voxels within the liver, kidney, and spleen on spectroscopic imaging. Artifacts were minimal, despite the high ^2^H_2_O concentration in the stomach immediately after ingestion, which can be problematic with the use of a volume coil.

**Conclusion:**

We have shown the feasibility of abdominal deuterium MR spectroscopic imaging at 3 T using a flexible surface coil. Water measurements were obtained in healthy volunteers, and images were acquired in a patient with a renal tumor after drinking ^2^H_2_O. The limited depth penetration of the surface coil may have advantages in characterizing early uptake of orally administered agents in abdominal organs, despite the high concentrations in the stomach which can pose challenges with other coil combinations.

## 1 INTRODUCTION

Deuterium metabolic imaging (DMI) is an emerging method that has shown great promise for noninvasive assessment of tissue metabolism. The technique was first implemented in humans at high field strength,^1^ with recent studies showing its feasibility at 3 T.^2^, ^3^ Although the technique has great potential for imaging metabolism outside of the brain, only a few clinical studies have undertaken in the abdomen.^1^, ^4^, ^5^ Extracranial DMI presents significant hurdles, which we have sought to address in this study.

Deuterium‐labeled probes, such as ^2^H‐glucose and ^2^H_2_O, are typically administered orally in humans. This poses a physiological problem when using a volume transmit coil for abdominal imaging, as acquisition from the organ of interest can be overwhelmed by the signal arising from the stomach, especially early after administration.^4^ Here we have used a surface transmit‐receive coil to minimize the signal from the stomach, while enabling coverage of other abdominal organs. We have compared posterior and lateral coil positions and assessed the kidney following oral ^2^H_2_O administration at 3 T.

The low frequency of deuterium (19.6 MHz at 3 T) poses several challenges. Electromagnetic interference (EMI) artifacts^6^ can pose significant problems for X‐nuclei, which are often not tested during system design and installation. Additionally, there can be a significant problem with eddy current correction for multinuclear species on some scanners,^7^ particularly at the low frequency of deuterium. Here we have shown images from healthy volunteers at natural abundance and from a patient with a renal tumor following oral administration of ^2^H_2_O.

## 2 METHODS

**FIGURE 1:**
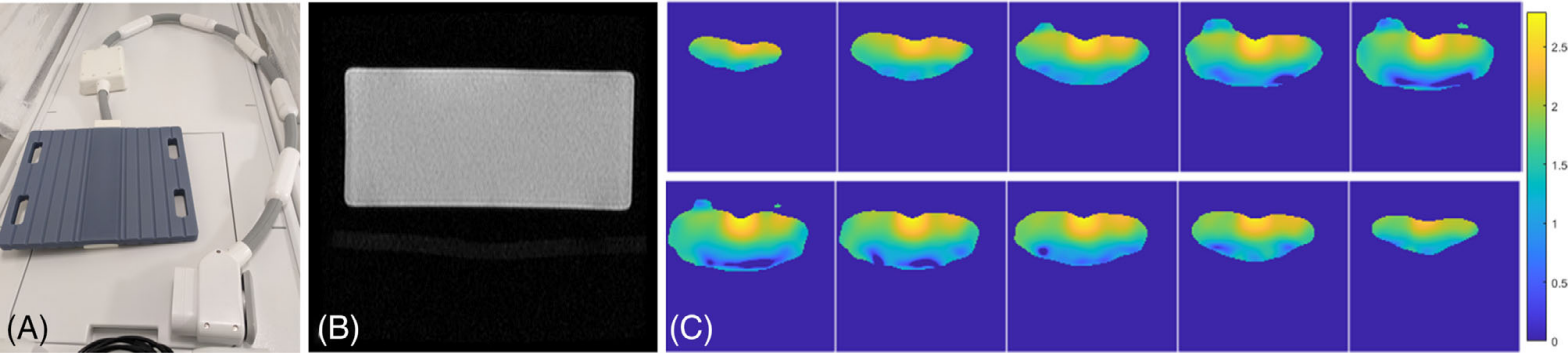
Double‐angle B_1_ maps for deuterium. (A) The deuterium surface coil, measuring 20 × 30 cm. (B) A structural ^1^H image of the central slice from a uniform rectangular phantom of silicone oil, dimensions = 32 × 22 × 16 cm, field of view = 40 cm. (C) A 10 × 10 × 10 matrix was acquired using three‐dimensional (3D) MR spectroscopic imaging with nominal flip angles of 60º and 30º. A 3D B_1_ map was calculated according to the double‐angle method and interpolated to 128 × 128 × 20. The central 10 slices are shown, covering the 20‐cm extent of the coil, which rested on top of the phantom. Intensities represent the ratio of actual to nominal flip angles.

Experiments were performed on a 3T Premier MR system (GE Healthcare, Waukesha WI, USA). Deuterium acquisition was performed with a flexible 20 × 30 cm transmit‐receive surface coil (Rapid Biomedical, Rimpar, Germany; Figure 1A). Structural ^1^H‐MRI was performed using the inbuilt body coil.

Signal strength and uniformity, including mapping of the B_1_^+^ field, were assessed using the naturally abundant deuterium signal within a 32 × 22 × 16 cm rectangular phantom filled with silicone oil (GE Healthcare). The severity of spectral distortion due to eddy currents was assessed by acquiring spectra at the minimum repetition time (TR) with no dummy scans^7^ (TR = 420 ms, spectral width = 5000 Hz, 2048 points, 8 transients). Acquisitions were performed in all three orientations in turn, to assess the effect of spoiler application along the X, Y, and Z directions.

Twelve human participants were included with written informed consent: 11 healthy volunteers (HeVoMRI study, Yorkshire & the Humber–Leeds East Research Ethics Committee number: 23/YH/0127) and 1 male patient in his 60s with a left‐sided, 5.2‐cm renal oncocytoma (IBM‐Renal,^8^ East of England–Cambridge East REC: 22/EE/0136). Histopathology was determined on surgical specimens post nephrectomy. Subjects were positioned supine with the deuterium coil placed either centrally underneath the subjects to target both kidneys (5 healthy volunteers and 1 patient) or wrapped around the right side of the abdomen and secured with Velcro straps, to target the liver and right kidney (*n* = 6).

An axial T_1_‐weighted dual gradient‐echo fat/water (LAVA‐flex) series was acquired in a single breath‐hold using the inbuilt ^1^H body coil (flip = 12°, echo time [TE] = 1.1/2.2 ms, TR = 4.0 ms, field of view [FOV] = 48 cm, matrix = 300 × 200, 64 × 5 mm–thick slices, one average, 167‐kHz full‐width receiver bandwidth) for the purpose of anatomical localization. Automated shimming on ^1^H was performed over a point‐resolved spectroscopy–selected voxel covering the approximate sensitive volume of the coil. Unlocalized ^2^H spectra were collected from the whole sensitive volume of the coil (TE = 0.7 ms, TR = 600 ms, flip = 90°, 64 averages, total duration = 39 s), followed by Hamming‐filtered density‐weighted MR spectroscopic imaging (MRSI) (matrix size = 16 × 16 × 16 nominal, actual = 10 × 10 × 10; 1678 transients, FOV = 40 cm, flip angle = 60°, TE = 1.3 ms, TR = 250 ms, total duration = 7 min; see Figure S1). A matching MRSI data set with flip 30° was acquired in 4 subjects for generation of a double‐angle method B_1_ map.^9^ Unlocalized spectra were repeated within the session in 2 subjects, and between sessions approximately 10 days apart in 5 subjects, with a further third session in 3 subjects. The patient fasted for 2 h before imaging and was administered a 5% ^2^H_2_O oral solution (190 g of sterile water for injection +11.1 g of ^2^H_2_O) immediately before the scan. Healthy subjects did not drink ^2^H_2_O or fast.

MRSI data were zero‐filled once in each spatial domain before automated peak fitting of the water and lipid signals using the *AMARES* package^10^ within a locally customized version of the OXSA spectroscopy toolbox.^11^ Voxels were excluded from fitting if the signal‐to‐noise ratio (SNR) of the maximum peak was below a threshold of 5. The largest peak was assigned to water at 4.7 ppm, and peaks were then fit for water and lipids using initial linewidth estimates of 10 and 20 Hz, respectively, with a maximum of 30 Hz. The value of ^2^H_total_ was calculated as the combined signal from ^2^H‐water and ^2^H‐lipids. The ratio of the ^2^H‐water to ^2^H_total_ amplitudes was compared in unlocalized spectra and between voxels selected from the liver, kidneys, and spleen. Voxels within the tumor and stomach were additionally compared in the patient. Voxels were excluded from further analysis if the Cramér‐Rao lower bounds (CRLBs) reported for the fitting of both peaks were above 10%. Overlays on anatomical images were created using *Radiant* 2023.1 (Medixant, Poznan, Poland).

## 3 RESULTS

### 3.1 System characterization and optimization

The Supporting Information details the characterization of EMI artifacts and spatial resolution.

Maps of the transmit B_1_ field in a large uniform phantom showed the expected pattern for a surface coil, with flip angles peaking at the center of the coil and decreasing rapidly with distance (Figure 1C). Good signal was observed up to a depth of approximately 12 cm.

**FIGURE 2:**
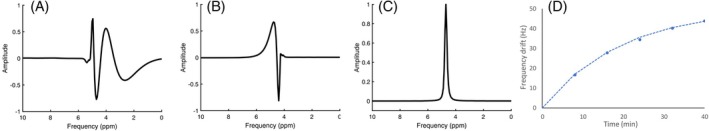
Demonstration of eddy current effects on deuterium data from different scanners and software versions. (A–C) Distortions in the spectral line shape as seen on a GE MR750 device at software level DV26 (A); a GE Premier scanner at software level MR29 (B); and the same Premier scanner at software level MR30, following the adoption into the product software of a fix for X‐nuclear eddy currents detailed in McLean et al.^7^ (C). (D) A significant drift in deuterium frequency over time was demonstrated in a static phantom of silicone oil acquired with the MR29 software, which may have been eddy current related as this drift was not present on MR30 software. An exponential fit to the data (*dashed line*) showed an apparent time constant of 19.4 min.

Although eddy current effects were greatly reduced on the Premier system in comparison to a previous GE MR750 system, they still produced distorted spectral line shapes when acquiring data at the minimum TR at the software version MR29 (Figure 2). This may also have resulted in a temporal frequency drift: Over the first 40 min, the resonant frequency shifted by over 40 Hz, in both the phantoms and in vivo, with an apparent time constant of 19.4 min. Following an upgrade to the MR30 software version, which included our suggested correction for the eddy current problem within the product system software, neither line shape distortions nor frequency drift were observed.

### 3.2 Human data

**FIGURE 3:**
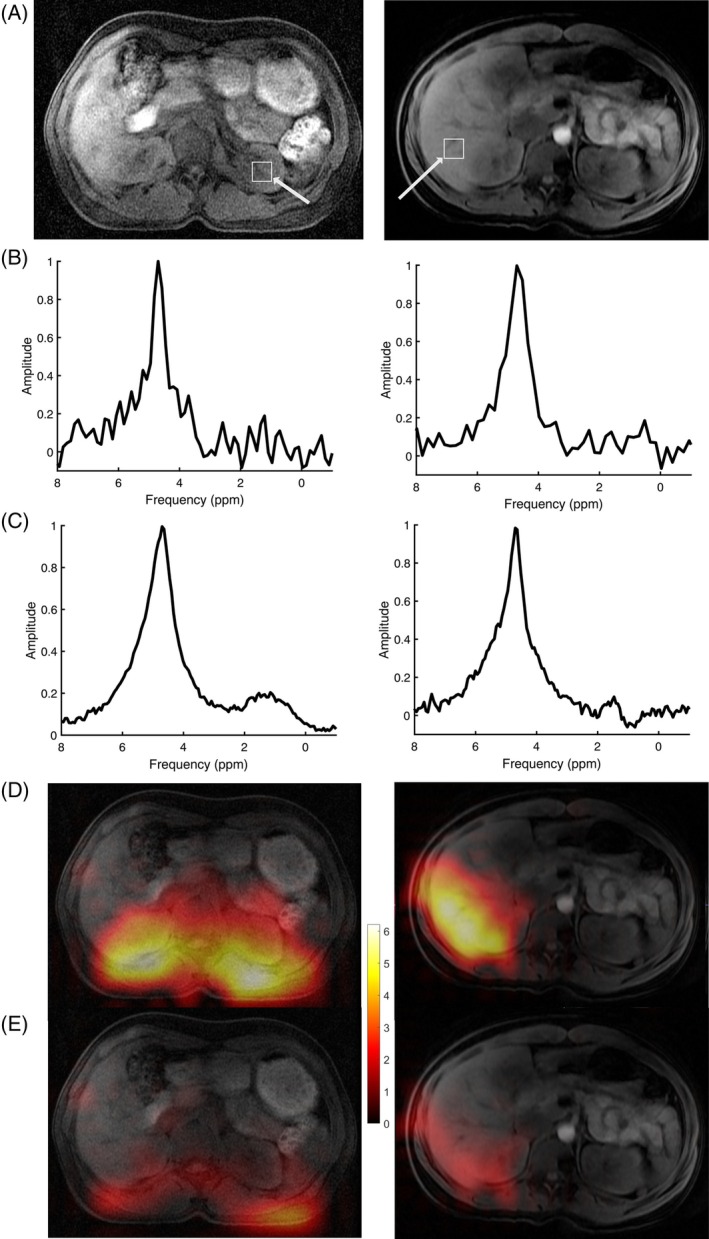
Comparison of data acquired with the deuterium coil placed under the lower back (*left column*) or wrapped around the right side of the abdomen (*right column*). (A) T_1_‐weighted image showing the location and size of the extracted MR spectroscopic imaging voxel in (B) from a grid interpolated once to 20 × 20 × 20 mm. (C) Spectra from unlocalized MR spectroscopy. (B,C) Zero filling and 1‐Hz Gaussian line broadening were applied for display. Water (D) and lipids (E) are shown overlaid on the images from (A). The color bar shows arbitrary intensities/10000.

Acceptable fits to unlocalized spectra were obtained in all cases (Table 1A and Figure 3; CRLBs for water ranged from 0.9%–3.2%, mean 1.6%). The CRLB for lipids was consistently higher than water, especially when the coil was placed laterally rather than posteriorly (mean CRLB 17% lateral vs. 5% posterior; *p* < 0.05). As expected, the linewidths of the lipid peaks were often larger than those of water and were frequently at, or near, the permitted threshold of 30 Hz. The water fraction (^2^H_water_/^2^H_total_) was typically higher when the coil was in the lateral rather than the posterior position, but this was not significantly different (0.86 ± 0.11 vs. 0.75 ± 0.07; *p* = 0.08). The standard deviation of repeated measures of the water fraction within the same session was small (σ = 0.008), but the variation between sessions was higher (average σ = 0.042, range 0.014–0.075), which may be secondary to inconsistencies in the coil placement.

**TABLE 1:** Natural abundance deuterium data acquired in healthy volunteers. (A) Unlocalized spectroscopy. (B) Organ‐specific ratios of water to total ^2^H signal from MR spectroscopic imaging.

A: MRS							
Subject number	Coil position	Hwater2Htotal2	SD of repeats	CRLB % water	CRLB % lipid	Linewidth water (Hz)	Linewidth lipid (Hz)
1	Posterior	0.80	N/A	0.9	4.5	24	30
2	Lateral	0.98	0.0077^a^	1.4	33	21	2.5
3	Posterior	0.78	N/A	1.4	4.7	29	22
4	Posterior	0.80	0.0078^a^	1.3	7.2	21	30
5	Posterior	0.65	N/A	2.0	3.1	30	20
6	Posterior	0.71	N/A	2.0	5.0	30	25
7	Lateral	0.85	0.058	2.0	16	19	30
8	Lateral	0.68	0.075	3.2	7.7	26	30
9	Lateral	0.92	0.022	1.5	20	27	30
10	Lateral	0.79	0.042	1.1	5.1	26	30
11	Lateral	0.92	0.014	1.2	20	20	30
Mean		0.81 ± 0.11	0.04 ± 0.02	1.6 ± 0.6	12 ± 10	25 ± 4	25 ± 8

B: MRSI							
Subject number	Coil position	Liver	Right kidney	Left kidney	Spleen	Repeats SD: liver	Repeats SD: kidney
1	Posterior	0.84	0.83	0.85	0.80	–	–
2	Lateral	0.86	0.76	–	–	–	–
3	Posterior	0.73	0.59	0.70	0.75	–	–
4	Posterior	0.98	0.80	0.86	0.86	–	–
5	Posterior	–	–	–	–	–	–
6	Posterior	0.75	–	0.71	0.58	–	–
7	Lateral	0.72	0.90	–	–	0.105	0.036
8	Lateral	0.77	0.58	–	–	0.032	0.046
9	Lateral	0.93	0.86	–	–	0.075	0.068
10	Lateral	0.84	0.75	–	–	0.035	0.056
11	Lateral	0.88	0.75	–	–	0.048	0.040
Mean		0.83 ± 0.09	0.76 ± 0.11	0.78 ± 0.09	0.75 ± 0.12	0.06 ± 0.03	0.05 ± 0.01

Abbreviations: CRLB, Cramér‐Rao lower bound; MRS, MR spectroscopy; MRSI, MR spectroscopic imaging; SD, standard deviation.

^a^ Denotes intrasession repeatability, as opposed to the intersession repeatability in later subjects.

Spectra from MRSI voxels within the liver, kidneys, and spleen also yielded acceptable fits when these organs were within the sensitive volume of the coil (Table 1B). An exception was Volunteer 5, in whom no voxels from the kidneys nor liver met the required SNR threshold, probably because the coil was placed too far inferior (Figure S4). Ratios of ^2^H_water_ /^2^H_total_ were consistent within and between organs and volunteers. The highest ^2^H_water_ fractions were seen in the liver, but comparisons with other organs did not reach significance (paired t‐test comparing liver to the right kidney: *p* = 0.06). Reliable measurements in the left kidney were only achievable with posterior coil placement, as expected, given the distance from the coil when wrapped around the right side of the abdomen. Water fractions in the left kidney tended to be higher than in the right, but paired measurements were only obtained in 3 subjects (*p* = 0.15). The standard deviation of repeated measures for the ^2^H_water_ fraction in the liver was 0.06 (range 0.03–0.11) and in the kidney was 0.05 (range 0.04–0.07).

**FIGURE 4:**
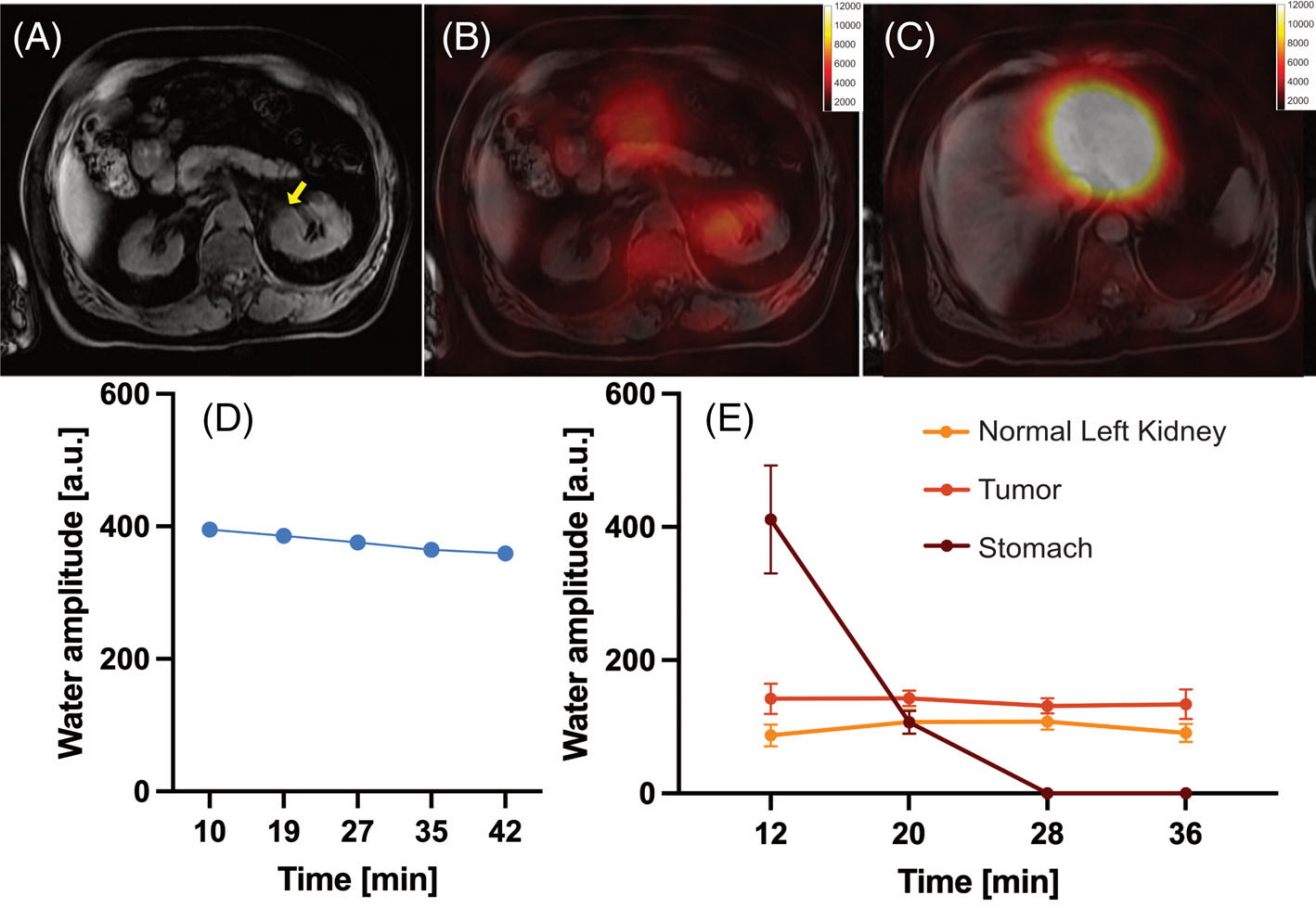
Images and signal acquired from a patient with a renal oncocytoma after drinking ^2^H_2_O. (A) Anatomical ^1^H water image from 2‐point Dixon three‐dimensional volume at the level of the tumor (*arrowed*). (B,C) Fitted water amplitude, zero‐filled to 128 × 128 and overlaid using the “hot” color scale on the anatomical proton images at the level of the tumor and stomach, respectively, 12 min after drinking the labeled water. (D) Water peak amplitude in the unlocalized spectra plotted against time from oral ingestion. (E) Water peak amplitude (mean and standard deviation) over all voxels within each organ where Cramér‐Rao lower bounds were < 10.

Significant stomach water signal was observed after ^2^H_2_O ingestion, despite the depth of the organ from the coil (13–25 cm; Figure 4). Although this initial signal was more intense than those from other organs, the distance from the coil mitigated the dynamic range difference, thus preventing severe point spread function artifacts; this effect resolved within 30 min. The tumor and contralateral kidney water signals remained relatively stable, while unlocalized water signal gradually decreased over 40 min.

## 4 DISCUSSION

Several technical challenges were overcome to allow implementation of deuterium imaging at 3 T in the human abdomen, both for the experimental setup and in characterizing and compensating for data quality issues.

Our GE Premier system exhibited less severe eddy currents than our previous MR750^7^ but still produced significant distortion of the spectral line shape (Figure 2). More surprisingly, a drift in deuterium frequency over time was observed, with an apparent time constant of about 20 min. We infer that this may have been due to eddy currents, because with an upgrade to a software version that incorporates our suggested eddy current correction, this frequency drift was eliminated along with the line shape distortions.

The use of a k‐space sampling scheme in the abdomen with a broad point spread function would be expected to cause severe problems with signal contamination of adjacent voxels from both abdominal fat and from the orally administered tracer within the stomach. However, as demonstrated in Figure 4, the strong stomach signal observed after ^2^H_2_O ingestion was effectively confined to that organ using a trajectory with a favorable point spread function (Figure S1). Furthermore, the signal from the renal tumor can be distinguished from the surrounding kidney, enabling potential quantification of differences between abdominal structures.

Despite their inhomogeneous sensitivity profiles, surface coils offer advantages for deuterium imaging, as demonstrated in human liver studies.^1^, ^12^ The high flip angles near the coil surface can be exploited to minimize signal from subcutaneous fat. In this study, the nominal flip angle of 60º resulted in an actual flip angle near 180º for subcutaneous fat, nulling the signal (Figure 3).

A key advantage of surface coils is the minimization of signal from the oral tracer within the stomach. According to the Biot‐Savart law, for a surface coil of radius *R*, signal at a depth *y* is expected to decline on‐axis as a function of (y^2^ + R^2^)^3/2^. Consequently, both receive coil sensitivity and transmit flip angles are approximately 4 times lower at the depth of the stomach (˜20 cm) compared with the kidney and tumor (˜10 cm), for an overall signal reduction of over 13‐fold. Even so, Figure 4 shows the stomach signal is more than twice that in the kidney and tumor, emphasizing the risk of signal swamping when a coil with a homogenous profile is used.

Intravenous administration of the labeled probe, either as a bolus or infusion, would eliminate the problem of hyperintense stomach signal associated with oral delivery. This approach has been used successfully in rodent studies of deuterium‐labeled probes in tumors and abdominal organs,^1^, ^13^, ^14^ and intravenous glucose administration is a safe and established procedure in human glucose tolerance tests for diabetes assessment.^15^ Although deuterium‐labeled glucose for human injection can be more expensive, potential dose reduction and broader adoption could mitigate the cost in the future. Nevertheless, oral administration remains ideal for routine clinical use.

An alternative method to reduce the influence of the hyperintense stomach signal involves extending the time interval between oral tracer administration and DMI data collection; however, intersubject variability in glucose absorption from the GI tract might complicate interpretation. For example, in 2 subjects with overlapping time courses, 1 showed glucose signal still rising in both liver and kidney 130 min after ingestion, whereas the other showed glucose signal in both organs was already falling 68 min after intake.^4^ These discrepancies could arise from differences in either the rate of glucose absorption or its subsequent metabolism: the deuterium label could be subsequently transferred into ^2^H‐glycogen, which is MR‐invisible,^16^ to ^2^H‐lactate though glycolysis, or ultimately into ^2^H‐water as part of oxidative metabolism.^17^, ^18^

Prolonged waiting times before measurement are particularly problematic when assessing lactate formation in tumors: as the lactate and lipid signals overlap and the timescale of lactate production following glucose administration is uncertain, observing its change over time is required for an accurate assessment. We have previously demonstrated using unlocalized whole head spectra in a healthy volunteer that there is a gradual rise in the peak identified at 1.35 ppm during the hour following glucose ingestion, whereas the fat signal at 0.9 ppm was largely unchanged.^2^ From this, it is reasonable to infer that an increase in the labelling of the lactate pool was the source of the signal elevation. Although it might be possible to observe a decline in such a peak some hours after administration due to lactate washout, the time course of that process is likely to be more variable and challenging to interpret.

In healthy volunteers, we recorded naturally abundant deuterium signals with the coil placed either posteriorly for equal sensitivity over both kidneys, or laterally to interrogate the liver and right kidney. A strong unlocalized signal was recorded in all subjects, with a water/fat ratio of around 4:1 on average. Spectral linewidths were approximately 25 Hz, suggesting that the glucose peak at 3.9 ppm would not be fully resolved from water at this field strength. However, our previous work demonstrated the robustness of spectral fitting using the OXSA spectroscopy toolbox for DMI at 3 T in the brain.^2^, ^18^ The within‐session repeatability of MRS was excellent, with a standard deviation of repeated measures below 1%. Between sessions, the repeatability was approximately 5% for unlocalized MRS, and about 7% for MRSI voxels derived from liver and kidney. Although our 7‐min MRSI sequence was generally robust, it did not detect sufficient signal from the kidneys and liver for reliable quantification in 1 subject (Subject 5), which was probably due to suboptimal coil placement (Figure S4). Nevertheless, the repeatability for organ‐specific averages was only slightly higher than for unlocalized spectra despite the limited SNR (Figure 3), supporting the feasibility of longitudinal assessments.

In the patient with a renal oncocytoma, the deuterium signal was higher in the tumor compared with the surrounding normal‐appearing kidney within 12 min of oral ingestion of ^2^H_2_O. Importantly, despite the region‐of‐interest location on the left side of the abdomen, the tumor signal was not overwhelmed by stomach signal as in previous DMI studies.^4^ Future work could focus on comparing signal contrast between benign and malignant renal masses due to the clinical importance of such differentiation and its effect on treatment decisions.^8^ Preclinical rodent studies suggest that the uptake of ^2^H_2_O can produce tumor contrast^19^ and potentially monitor cancer therapy response.^20^ The challenges in distinguishing ^2^H‐lactate from ^2^H‐lipid might render DMI approaches for assessing glycolytic metabolism using [6,6′‐^2^H_2_]glucose difficult in the abdomen. In comparison, the use of ^2^H_2_O as a cheap and simple contrast agent could provide an interesting alternative for assessing tumor perfusion and diffusion. Fitting of the water peak is relatively straightforward and the absorption of water from the gut is much more rapid, reproducible, and less dependent on fasting state than for glucose.^21^ This approach could be complementary to conventional methods for assessing tumor perfusion such as the use of much larger gadolinium‐based contrast agents.

## 5 CONCLUSIONS

We have demonstrated the feasibility of imaging naturally abundant deuterium in the human abdomen at 3 T. Water and lipid peaks were fit repeatably and with high confidence both from unlocalized spectra and from MRSI voxels within the liver, kidneys, and spleen. Consistent ^2^H_water_/^2^H_total_ ratios were observed within and between sessions. The coil configuration and pulse sequence used were shown to avoid excessive point spread artifact from the stomach signal, even within 12 min after the oral administration of a high dose of ^2^H_2_O. Heavy water may be an interesting probe for tumor characterization and response monitoring, and the current work establishes the background signal that could be observed in future studies using oral deuterated glucose at clinical field strength in cancer and other diseases.

## CONFLICT OF INTEREST

Rolf Schulte is an employee of GE Healthcare.

## Supporting information


**Figure S1.** Density‐weighted MR spectroscopic imaging (MRSI) scheme used for acquisition of deuterium metabolic imaging data. (A) Distribution in k‐space of the 1678 samples. (B) Calculated point‐spread function in each dimension: The asterisks show the full width at 64% of maximum height, which is 0.94. (C) Magnitude of the predicted point spread function in the axial, sagittal, and coronal directions (*left to right*).
**Figure S2.** Images of peak ^2^H_2_O signal intensity derived from MR spectroscopic imaging (MRSI; *hot color scale*) overlaid on a three‐dimensional (3D) gradient‐echo structural image of the QalibreMD phantom in black and white. (A–C) Sections are shown in the axial (A), coronal (B), and sagittal (C) orientations, demonstrating that a shift of the deuterium image toward the right (patient left) is needed to optimally align with the structural image. MRSI was acquired using the same orientation, coil, and trajectory used in the current study.
**Figure S3.** (A,B) Deuterium power spectra acquired with the excitation pulse power set to zero to demonstrate electromagnetic interference. (A) Acquisition over a spectral bandwidth of 125 kHz reveals extensive artifact peaks in the frequency domain. (B) Reducing the bandwidth to 5 kHz, as used for the experiments in vivo, demonstrates a lack of significant interference in the center of the spectral range, and a decrease in the maximum peak scale by a factor of approximately 10^4^. (C) Unlocalized spectrum collected from Volunteer 1, with 64 transients, displaying the absolute value of the sum of the phase‐cycled transients. (D) Same spectrum with the sum taken of the absolute value of each transient, to eliminate the effect of phase cycling and maximize the electromagnetic interference (EMI) artifacts.
**Figure S4.** (A,B) Overlays on anatomic images of water (A) and fat signals (B) fitted from MR spectroscopic imaging (MRSI) data from Volunteer 5, with posterior coil placement (units are arbitrary intensity divided by 10 000).
