# Supplementary material for: Development and optimization of human deuterium MR spectroscopic imaging at 3 T in the abdomen
Source: Magn Reson Med. 2025 May 20;94(4):1377–85. doi: 10.1002/mrm.30556 (PMC12309867; doi:10.1002/mrm.30556)
Supplement: Supplementary file 1 — Figure S1. Density‐weighted MR spectroscopic imaging (MRSI) scheme used for acquisition of deuterium metabolic imaging data. (A) Distribution in k‐space of the 1678 samples. (B) Calculated point‐spread function in each dimension: The asterisks show the full width at 64% of maximum height, which is 0.94. (C) Magnitude of the predicted point spread function in the axial, sagittal, and coronal directions (left to right). Figure S2. Images of peak 2H2O signal intensity derived from MR spectroscopic imaging (MRSI; hot color scale) overlaid on a three‐dimensional (3D) gradient‐echo structural image of the QalibreMD phantom in black and white. (A–C) Sections are shown in the axial (A), coronal (B), and sagittal (C) orientations, demonstrating that a shift of the deuterium image toward the right (patient left) is needed to optimally align with the structural image. MRSI was acquired using the same orientation, coil, and trajectory used in the current study. Figure S3. (A,B) Deuterium power spectra acquired with the excitation pulse power set to zero to demonstrate electromagnetic interference. (A) Acquisition over a spectral bandwidth of 125 kHz reveals extensive artifact peaks in the frequency domain. (B) Reducing the bandwidth to 5 kHz, as used for the experiments in vivo, demonstrates a lack of significant interference in the center of the spectral range, and a decrease in the maximum peak scale by a factor of approximately 104. (C) Unlocalized spectrum collected from Volunteer 1, with 64 transients, displaying the absolute value of the sum of the phase‐cycled transients. (D) Same spectrum with the sum taken of the absolute value of each transient, to eliminate the effect of phase cycling and maximize the electromagnetic interference (EMI) artifacts. Figure S4. (A,B) Overlays on anatomic images of water (A) and fat signals (B) fitted from MR spectroscopic imaging (MRSI) data from Volunteer 5, with posterior coil placement (units are arbitrary intensity divided by 10 000) [file MRM-94-1377-s001.docx]

Supplementary information for:

Development and optimization of human deuterium MRSI at 3 T in the abdomen

Mary A McLean, Ines Horvat Menih, Pascal Wodtke et al.

Submitted to *Magnetic Resonance in Medicine*

**Section 1: Spatial resolution**

The Hamming-filtered density-weighted trajectory used for MRSI was designed with a nominal matrix size of 16 x 16 x 16, to give a true matrix size of 10 x 10 x 10 (22). Its favorable point spread function was demonstrated using simulations in MATLAB (Fig. S1).

Spatial resolution and registration with structural images were assessed experimentally using the Qalibre System Standard Model 130 phantom (QalibreMD, Boulder, CO, USA), a 20 cm inner diameter sphere of deionized water (H_2_O) which included a plate with a ring of 14 spheres (each with inner diameter 1 cm) containing varying proportions of ^2^H_2_O and ^1^H_2_O (deuterium percentage 0, 10, 20, 30, 40, 50, 60, 65, 70, 75, 80, 85, 90, 95%).

Although the actual matrix size collected was only 10 x 10 x 10, good spatial resolution could be demonstrated in this phantom (Fig. S2). It was also apparent that a small shift in the left-right direction was needed to align with the structural ^1^H images: this was applied manually in human data presented here. The source was later identified to be a bug in reconstruction and has been fixed.


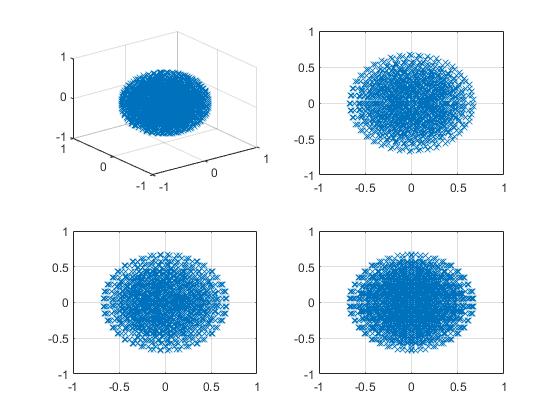

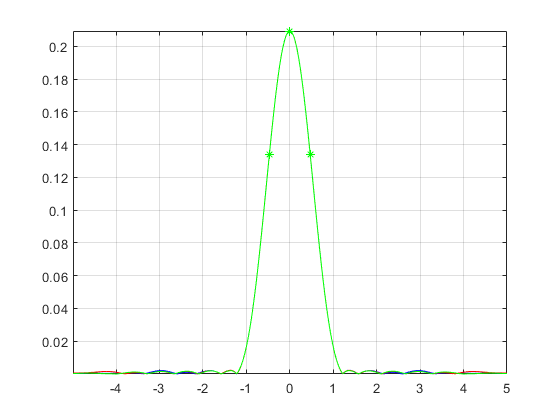

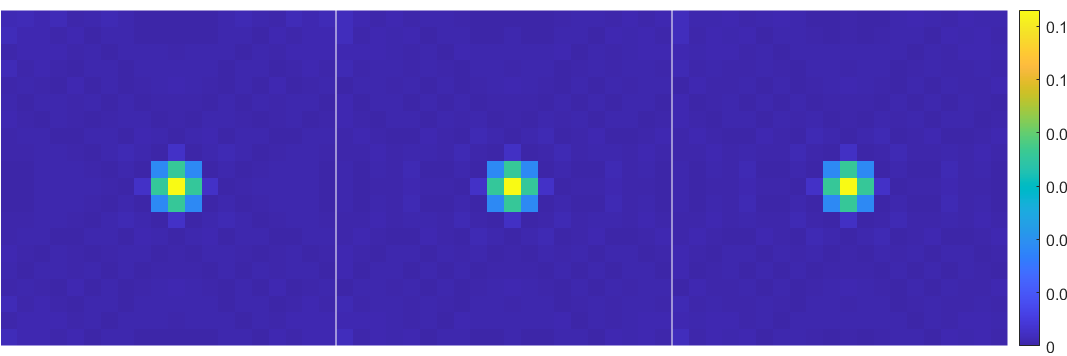


A

C

B

**Figure S1**: Density-weighted MRSI scheme used for acquisition of deuterium metabolic imaging data. (A) Distribution in k-space of the 1678 samples. (B) Calculated point-spread function in each dimension: the asterisks show the full width at 64% of maximum height which is 0.94. (C) Magnitude of the predicted point spread function in the axial, sagittal and coronal directions (left to right).


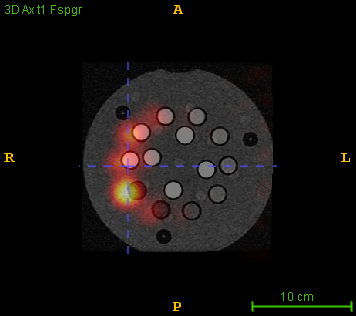

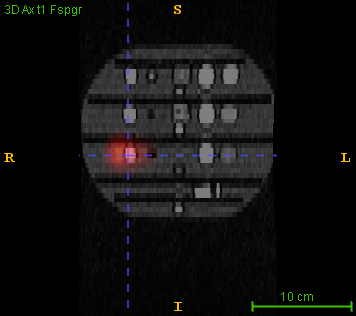

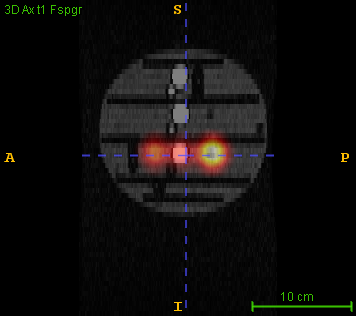


C

A

B

**Figure S2**: Images of peak ^2^H_2_O signal intensity derived from MRSI (hot colorscale) overlaid on a 3D gradient echo structural image of the QalibreMD phantom in black and white. Sections are shown in the (A) axial, (B) coronal, and (C) sagittal orientations demonstrating that a shift of the deuterium image toward the right (patient left) is needed to optimally align with the structural image. MRSI was acquired using the same orientation, coil, and trajectory used in the current study.

**Section 2: Electromagnetic interference (EMI)**

The severity of EMI at the deuterium frequency was assessed by acquiring spectra with the amplitude of the excitation pulses set to zero. Power spectra with 128 averages were plotted with the following sets of acquisition parameters: spectral bandwidth 125,000 Hz, 8192 points, TR 88.7 ms; bandwidth 5000 Hz, 2048 points, TR 419.6 ms (Fig. S3 A & B)

The deuterium coil detected many very intense RF spikes in the neighbourhood of the deuterium frequency range due to EMI; however, the 4000 Hz range centered around the resonant frequency of ^2^H_2_O was free of significant artifacts. The regular pattern of spikes, with the largest ones recurring at intervals of approximately 4000 Hz, suggested an electrical source, but this could not be identified as arising from the lighting or machinery within the scan room. The Premier system includes improvements in gradient filters previously described (3).

The magnitude of EMI peaks in vivo was also assessed in unlocalized spectra acquired as described in the main paper. The largest EMI peak detected in vivo was at -270 Hz relative to the water peak, well outside the frequency range of interest. In a phase-cycled unlocalized spectrum with 64 transients, it was undetectable. However, if the magnitudes of each transient were combined, this peak’s height was about 20% of the height of the water peak. Baseline offset is also visible in this display, which phase cycling effectively eliminates.

Large EMI peaks overlapping the spectral peaks of interest would prevent deuterium imaging and this may be problematic on some systems. Possible solutions include identifying and removing or filtering the sources of the EMI artifacts or ramping the magnet up or down to change the B_0_ field strength and therefore shifting the resonant frequency of deuterium away from the frequencies of the largest artifacts. However, the latter approach could potentially create problems for other nuclei of interest. It should be noted that deuterium imaging techniques which use gradient readouts over a large bandwidth acquisition (e.g. 20 kHz for balanced steady state free precession compared to 2.9 kHz for MRSI (23,24) are expected to be unworkable on a system which is prone to severe EMI artifacts.

**
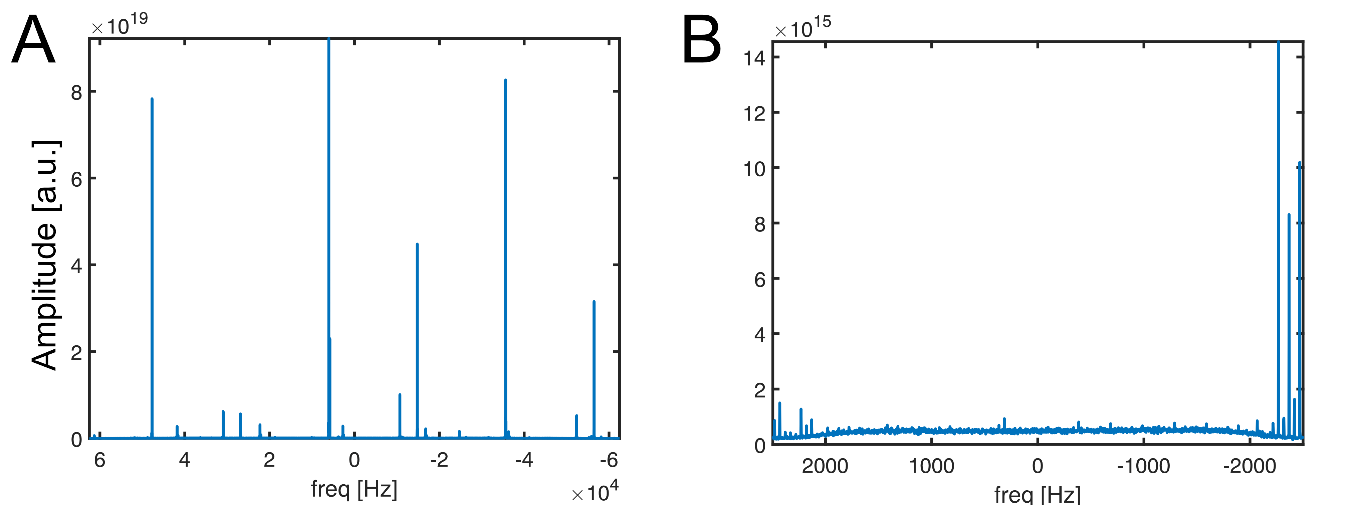
**

| **C** | **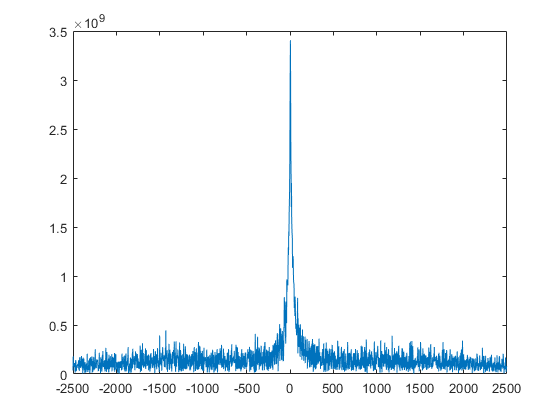** | **D** | **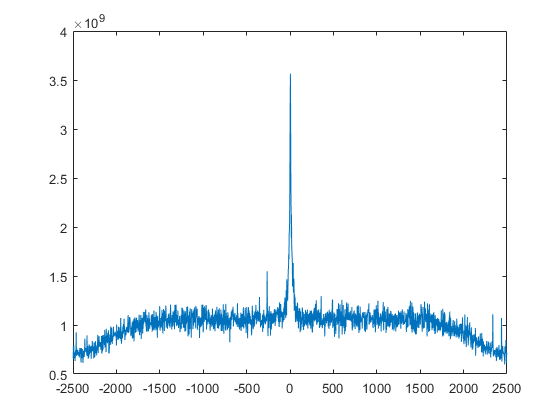** |
| --- | --- | --- | --- |

**Figure S3** (A & B) Deuterium power spectra acquired with the excitation pulse power set to zero to demonstrate electromagnetic interference. (A) Acquisition over a spectral bandwidth of 125 kHz reveals extensive artifact peaks in the frequency domain. (B) Reducing the bandwidth to 5 kHz, as used for the experiments *in vivo*, demonstrates a lack of significant interference in the center of the spectral range, and a decrease in the maximum peak scale by a factor of approximately 10^4^. (C) Unlocalized spectrum collected from volunteer 1, with 64 transients, displaying the absolute value of the sum of the phase-cycled transients. (D) Same spectrum with the sum taken of the absolute value of each transient, to eliminate the effect of phase cycling and maximize the EMI artifacts.

C

A

B

**Section 3: MRSI failure in Subject 5**

Although good water signal was detected from the paraspinal muscles, signal from the kidneys was insufficient for the quantification threshold of CRLB < 10%. In retrospect, the coil placement was too far toward the inferior, with the middle of coil at the level of the inferior pole of the kidneys, so it should have been repositioned further up. Alternatively, lateral placement of the coil may have yielded sufficient liver signal since the layer of subcutaneous fat is thinner over the ribs compared to the lower back and the liver is a more superficial organ compared to the kidneys. Since this subject had the highest BMI, it is also possible that a higher transmit power would have improved the signal, instead of relying on an automated Bloch-Siegert prescan which may have been biased toward more superficial tissues. Signal could also have been improved with longer scan times: collecting 2 averages in 14 minutes would remain clinically acceptable. However, coil penetration is an obvious limitation of this surface transmit-receive design, particularly in larger subjects.

| 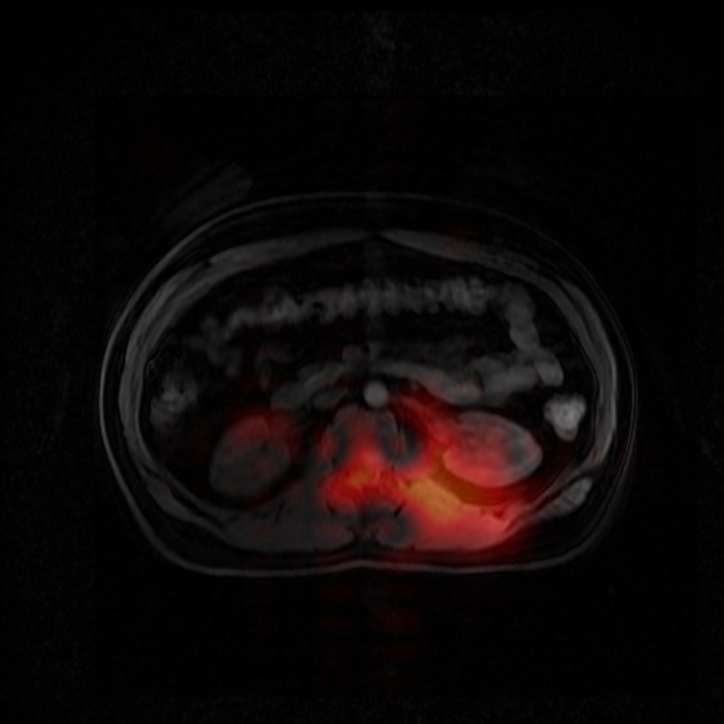  A | 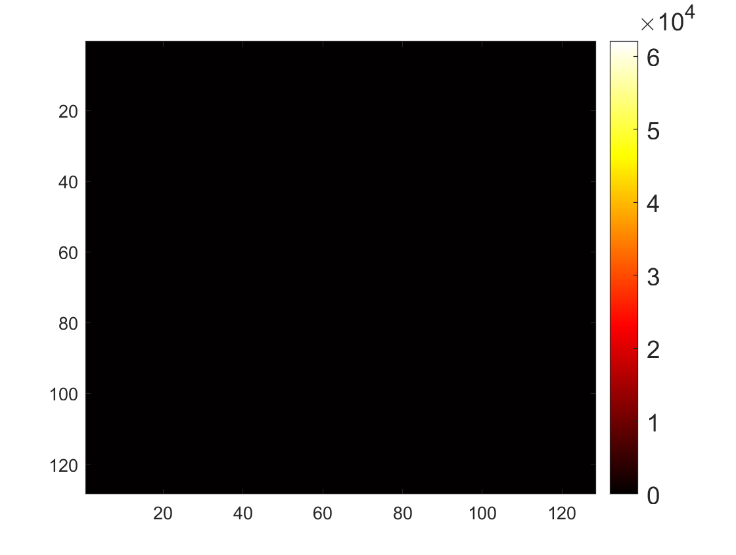 | 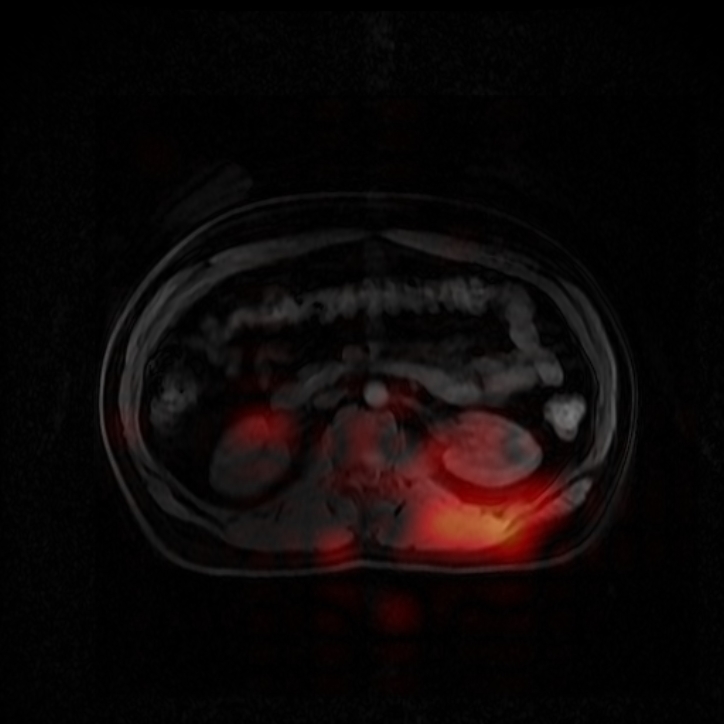  B |
| --- | --- | --- |

**Figure S4**: Overlays on anatomic images of (A) water and (B) fat signals fitted from MRSI data from Volunteer 5, with posterior coil placement (units are arbitrary intensity divided by 10,000).

**Additional References**

22. Greiser A & von Kienlin M. Efficient k-space sampling by density-weighted phase-encoding. Magn Reson Med 2003; 50(6):1266-75.

23. Peters DC, Markovic S, Bao Q, et al. Improving deuterium metabolic imaging (DMI) signal-to-noise ratio by spectroscopic multi-echo bSSFP: a pancreatic cancer investigation. Magn Reson Med 2021; 86:2604-2617.

24. Montrazi ET, Sasson K, Agemy L, et al. High-sensitivity deuterium metabolic MRI differentiates acute pancreatitis from pancreatic cancers in murine models. Sci Rep 2023; 13: 19998.
